# Polymerase chain reaction combined with fluorescent lateral flow immunoassay based on magnetic purification for rapid detection of canine parvovirus 2

**DOI:** 10.1186/s12917-019-1774-3

**Published:** 2019-01-17

**Authors:** Linlin Zhuang, Yongxin Ji, Peilong Tian, Kaixuan Wang, Chengkun Kou, Ning Gu, Yu Zhang

**Affiliations:** 1 State Key Laboratory of Bioelectronics, Jiangsu Key Laboratory for Biomaterials and Devices, School of Biological Sciences and Medical Engineering and Collaborative Innovation Center of Suzhou Nano Science and Technology, Southeast University, No. 2, Sipailou, Xuanwu District, Nanjing, Jiangsu Province 210096 People’s Republic of China; 2 Nanjing Nanoeast Biotech Co., Ltd., Nanjing, Jiangsu 210009 People’s Republic of China

**Keywords:** Canine parvovirus, Polymerase chain reaction, Fluorescent lateral flow immunoassay, Magnetic purification

## Abstract

**Background:**

Canine parvovirus 2 (CPV-2) is one of the most common etiological agents that cause severe gastroenteritis in puppies. Early accurate diagnosis is important for infected dogs. In recent years, magnetic separation has become an efficient and useful tool for bioassays. In this study, polymerase chain reaction (PCR) combined with fluorescent lateral flow immunoassay (LFIA) based on magnetic purification assay was developed for the quantitative detection of CPV-2.

**Results:**

The optimum working reaction volume and reaction time for LFIA was 100 μL and 2 min, respectively. The PCR-LFIA assay only detected CPV-2, and did not show cross-detection of non-CPV strains. Experiments showed analytical sensitivity of 3 × 10^1^ copies/μL and demonstrated the PCR-LFIA has a diagnostic agreement of 100% with conventional PCR on detection of clinical samples (22.6% positive, 14/62). Cutoff value is 146. The results were further verified by sequencing and BLAST software. The entire process from PCR step only takes ~ 80 min.

**Conclusions:**

This approach provides an attractive platform for rapid and quantitative detection of CPV-2, indicating great promise as a convenient molecular detection tool to facilitate disease outbreak investigations and response timely.

## Background

Dogs as the most popular pets have spread globally, leaving a tremendous ecological paw print and market potentiality [1]. In the world wide, there is an increasing interest in keeping of dogs for various reasons hence it is necessary to prevent important fatal infectious diseases of dogs [2].

Canine parvovirus (CPV), a member of the *Parvovirus* genus, is one of the most common etiological agents that cause severe gastroenteritis in puppies of mainly 6–20 weeks old, especially unvaccinated puppies or those with poor maternal protection through passive immunity [3, 4]. Parvovirus replicates mainly in intestinal crypts and characterized by being highly contagious. CPV is a variant of the feline panleukopenia virus (FPV) with the new nomenclature of canine parvovirus type 2 (CPV-2) and differs genetically and antigenically from the canine minute virus, which designed as CPV-1 [5]. CPV-2 is responsible for hemorrhagic gastroenteritis with high rates of mortality and morbidity and became widespread since 1978 [6]. From the outbreak of CPV-2, three new antigenic variants were characterized and termed as CPV-2a, CPV-2b and CPV-2c [7]. However,the clinical diagnosis of CPV-2 infection is indecisive, since several other pathogens may cause diarrhoea in dogs [8]. Therefore, early accurate diagnosis of CPV-2 infection is quintessential, so that the infected dogs can receive timely and effective treatment to reduce morbidity and mortality.

Specific and sensitive laboratory diagnostic methods available for CPV detection have been developed over the years. Routinely, immunological-based methods such as enzyme-linked immunosorbent assay (ELISA), immunochromatographic (IC) and haemagglutination (HA) have been used to screen faeces from diarrhoeic dogs, but these methods are affected by relatively low sensitivity [9–11]. Virus isolation (VI) is more sensitive and accurate, while it is too time-consuming and laborious for pathogen detection [12]. With advances in molecular techniques, conventional and real-time PCR assays were gradually developed for CPV-2 detection with higher sensitivity and specificity [13–15]. And further study showed that among above methods for detection of CPV-2, the best correlation was found between conventional and real-time PCR [8]. However, real-time PCR has not spread to the primary detection method due to its high equipment and reagent cost. Alternatively, conventional PCR assay based on agarose gel electrophoresis (PCR-GE), followed by nucleic acid dyes (such as ethidium bromide) staining, which poses a great potential threat to the health of the experimenter. All of these factors may have limited suitability for wide application.

To avoid gel electrophoresis and provide a portable, cost-effective platform to record results, PCR combined with lateral flow immunoassays (LFIAs), such as gold nanoparticle (AuNP)-based LFIAs, have been conducted [16–18]. PCR-LFIAs are convenient, quick and easy-operating. However, primer dimers and hairpins are known features in molecular diagnostic assays [19, 20]. Due to the primer as a modified carrier, the dimer formed by the upstream and downstream primers also has the characteristic of PCR amplification products, which can also be recognized as a positive by the LFIA. To overcome the obstacles of false positive result, researchers have been exploring some effective ways to address primer dimers to increase the accuracy and specificity [21, 22]. The probe method can reduce the effect of primer dimers, but it is still based on the principle of nucleic acid hybridization and difficult to solve the problem of primer (probe) dimers fundamentally. Furthermore, it not only adds the complexity of design and operation, but also increases inspection cost and time. On the other hand, the nucleic acid denaturant-based methods have great influence on DNA amplification products and the detection limit is affected. There is an urgent need in improved method for PCR products processing to higher accuracy.

In recent years, magnetic separation has become an interesting and useful tool for bioassays because the magnetic beads enable the isolation or extraction of target molecule or substance under the action of an external magnetic field [23, 24]. Due to the good biocompatibility and adequate functional groups for chemical fixation, magnetic beads modified with various recognition elements can be used for specific bioaffinity capture of different molecules. They are employed not only for magnetic separation of support from the reaction mixture but also as the solid adsorbent [25]. Therefore, magnetic beads have been applied to other areas such as immobilization of proteins and enzymes [26], bioseparation [27], immunoassay [28], drug delivery [29] and biosensors [30].

**Fig. 1:**
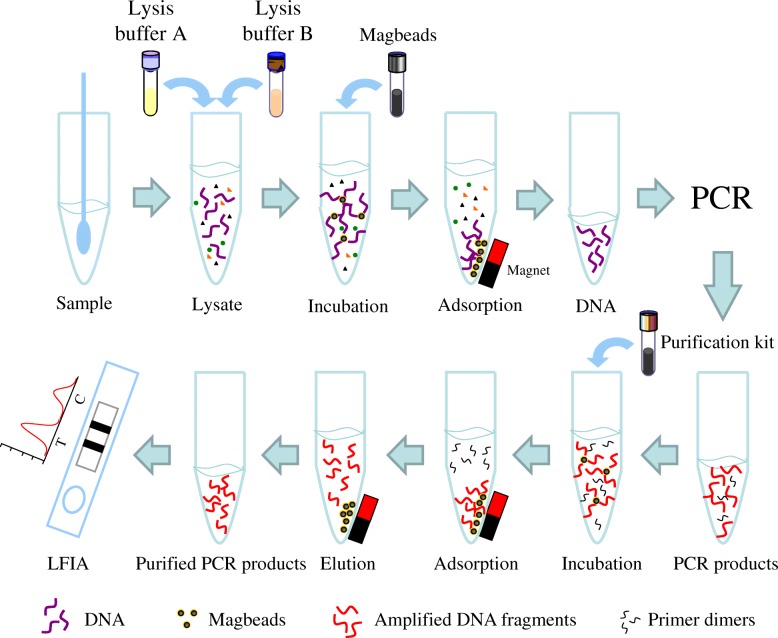
The illustration of PCR-LFIA for detection of CPV-2

In this study, we describe the development and validation of a sensitive and specific PCR method combined with fluorescent lateral flow immunoassay (PCR-LFIA) based on magnetic beads purification for rapid detection of CPV-2 (Fig. 1). The LFIA used nanoparticles as signal amplifier, which ensures the sensitivity and accuracy to replace gel electrophoresis. With the LFIA reader Nanoeasy 1700, results can be quantified and shared in real time via the internet. The PCR-LFIA assay is rapid, sensitive, specific and safe, indicating great promise as a convenient tool to facilitate disease outbreak investigations and response timely.

## Methods

### Virus strains and clinical samples

Three canine parvovirus positive strains were isolated previously and confirmed by DNA sequencing (CPV-2-N1 strain, CPV-2a, GenBank accession number: MH660523; CPV-2-N2 strain, CPV-2b, GenBank accession number: MH660524; CPV-2-N3 strain, CPV-2c, GenBank accession number: MH660525) [3]. Several non-CPV strains, including pseudorabies virus (PRV, PRV-R1 strain, GenBank accession number: MH727488), canine distemper virus (CDV, CDV-NJ2 strain, GenBank accession number: MH727489), canine coronavirus (CCoV, CCoV-C5 strain, GenBank accession number: MH727490), canine parainfluenza virus (CPIV, CPIV-J2 strain, GenBank accession number: MH727491) were maintained in our laboratory.

Sixty-two nasal/oropharyngeal and faecal swabs were collected from 51 dogs of both sexes (various breeds and ages) from the animal hospital of Jubilancy, Nanjing and 11 dogs from the farms in Jiangsu Province, China during 2018 and snap-frozen for storage at − 80 °C. These swabs were collected from diarrheic dogs and nine of them were suspected for CPV infection. All samples were collected using sterile cotton swabs and were immersed immediately in screw capped polypropylene tubes containing sterile PBS of pH 7.2 [31].

### DNA/RNA extraction

The swabs were squeezed and the liquid was extracted using the Magnetic Viral DNA Kit (Nanoeast, Nanjing, China) according to manufacturer’s instructions. Viral RNA of non-CPV strains was extracted using Trizol Reagent (TIANGEN, Beijing, China) according to manufacturer’s instructions. The concentration of DNA templates was quantified by Nano-300 Micro Spectrophotometer (Allsheng, Hangzhou, China). All DNA/RNA templates were stored at − 80 °C till further use.

### PCR conditions

The polymerase chain reaction (PCR) primers were especially designed from consensus genome regions of VP2 gene according to previous study [32]. The biotin-labeled forward primer was 5’-Biotin-AATGTACCACCAGTTTATC-3′ and the digoxigenin-labeled reverse primer was 5′-digoxigenin-TGGGAGGCTCTTAGTTTAG-3′. The primers were assessed using NUPACK web software (http://www.nupack.org/) and their specificity was further verified by BLAST tool (http://blast.ncbi.nlm.nih.gov/Blast.cgi). The upstream and downstream primers are located in the conserved region of the VP2 gene and reasonably avoid the mutation sites [33]. Primers were synthesized by GENEWIZ (GENEWIZ, Suzhou, China).

Polymerase chain reaction was set up by adding 10 μL Premix Taq™ (TaKaRa Taq™ Version 2.0) (TaKaRa, Dalian, China), 1 μL PCR primers (10 μM each), 1 μL DNA template and the reaction volume was made up to 20 μL using nuclease free ddH_2_O. PCR amplification was performed with an initial denaturation step of 94 °C for 5 min, 35 cycles of denaturation at 94 °C for 20 s, annealing at 50 °C for 20 s, and elongation at 72 °C for 20 s, followed by a final extension step of 72 °C for 8 min. PCR products were analyzed by 1.5% (*w*/*v*) agarose gel electrophoresis with DL-2000 DNA Marker (TaKaRa, Dalian, China) to estimate the fragment size of 253 bp. Finally, the PCR products were purified with Magbeads PCR Purification Kit (Nanoeast, Nanjing, China) for genomic sequencing (GenScript, Nanjing, China). And all CPV field strains detected in the clinical samples were characterised to assess the antigenic type according to previous report [33].

### PCR products purification and LFIA detection

PCR products were purified by Magbeads PCR Purification Kit (Nanoeast, Nanjing, China). According to manufacturer’s instructions, 5 μL of amplified DNA was mixed with 10 μL magbeads reagent (1:2). This ratio allows for optimal selection of PCR products with fragments length greater than 100 bp. The effect of PCR products purification was simultaneously verified by 1.5% (*w*/*v*) agarose gel electrophoresis and fluorescence lateral flow immunoassay (Nanoeast, Nanjing, China).

The final reaction volume for LFIA was optimized by adding the running buffer of 40 μL, 60 μL, 80 μL, 100 μL and 120 μL to the sample tube containing about 20 μL eluant, followed by mixing for 10 s. Then, the mixed solution was added to the sampling window of the fluorescent lateral flow strip. The reaction time was optimized by incubating the mixture for 60 s, 90 s, 120 s, 150 s, 180 s at room temperature. Finally, the images of test (T) and control (C) lines were recorded by fluorescence imager (Nanoeast, Nanjing, China) and the related fluorescent signal values were read by LFIA reader Nanoeasy 1700 (Nanoeast, Nanjing, China).

### Specificity of the PCR-LFIA

The specificity of the PCR-LFIA was established by testing three known positive strains (CPV-2-N1, CPV-2-N2, and CPV-2-N3) and several non-CPV strains (PRV-R1, CDV-NJ2, CCoV-C5, CPIV-J2) maintained in our laboratory. Previous study has verified the specificity of the PCR method based on its specific primers, the focus here is on verifying the specificity of PCR-LFIA [32]. Viral RNA was translated to cDNA by PrimeScript™ RT Master Mix (TaKaRa, Dalian, China) before PCR. Each sample test was repeated three times.

### Sensitivity of the PCR-LFIA

The genomic DNA of CPV-2-N1 strain was extracted for PCR amplification. PCR products were purified by Magbeads PCR Purification Kit (Nanoeast, Nanjing, China). The PCR amplification products were ligated into the pUC57 vector by using Hieff Clone™ Zero TOPO-TA Cloning Kit (Yeasen, Shanghai, China) after purified and cloned in *Escherichia coli*. Positive clones were identified by sequencing (GenScript, Nanjing, China). The positive clones were cultured and the plasmids were extracted using Magnetic Plasmid Extraction Kit (Nanoeast, Nanjing, China), and the content of the plasmids was determined by Nano-300 Micro Spectrophotometer, then the plasmids were stored at − 20 °C. The plasmid copy number was calculated following the formula [34]: copies/μL = (6.02 × 10^23^) × (ng/μL × 10^− 9^) / (DNA length × 660) by using the plasmid pUC57 containing the desired fragment as a standard. Standard DNA is diluted from 3 × 10^8^ to 3 × 10^0^ copies/μL for PCR amplification to evaluate the detection limit of PCR-LFIA.

## Results

### Optimization of the LFIA

**Fig. 2:**
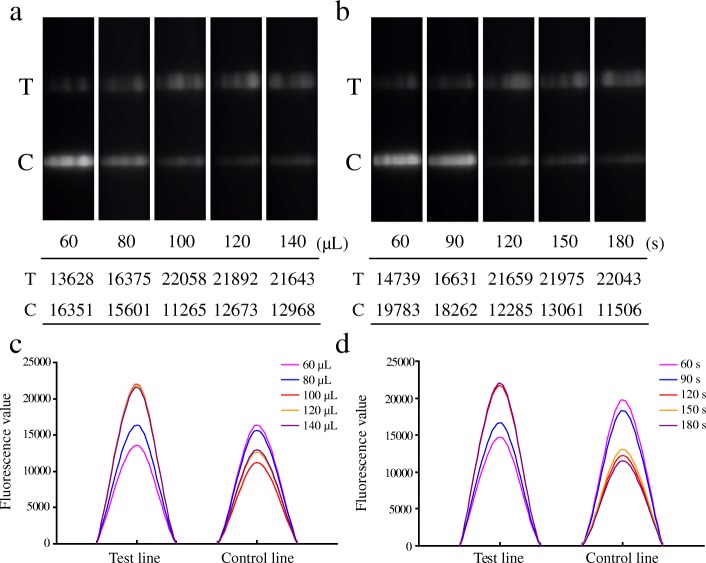
Assay condition optimization for LFIA. **a** The working reaction solutions containing the purified PCR products (20 μL) and running buffer with different volumes of 40 μL, 60 μL, 80 μL, 100 μL and 120 μL was tested. **b** The reaction time was optimized by adding 100 μL working reaction solution and incubating for 60 s, 90 s, 120 s, 150 s, 180 s at room temperature. The images of test (T) and control (C) lines were recorded by fluorescence imager. The fluorescence signals of test and control lines in **c** and **d** correspond to Fig. 2a and b, respectively

Purified PCR products were tested using the LFIA to determine the optimal final reaction volume and time (Fig. 2). To achieve maximal fluorescence signal on test line, we optimized the working reaction volume in the range of 60 μL–140 μL. As shown in Fig. 2, with the increasing of working reaction volume, the fluorescence signals reached the highest point in 100 μL and maintained stably till 140 μL (Fig. 2a and c). In addition, the fluorescence signals reached the highest point with incubating for 120 s at room temperature and slight difference in band clarity was observed with increasing reaction time from 120 s to 180 s (Fig. 2b and d). The results showed that the best reaction condition was 100 μL working reaction volume incubating for 120 s (2 min) at room temperature, and thus was chosen for the following experiments.

### Establishment of PCR-LFIA for CPV-2 detection

**Fig. 3:**
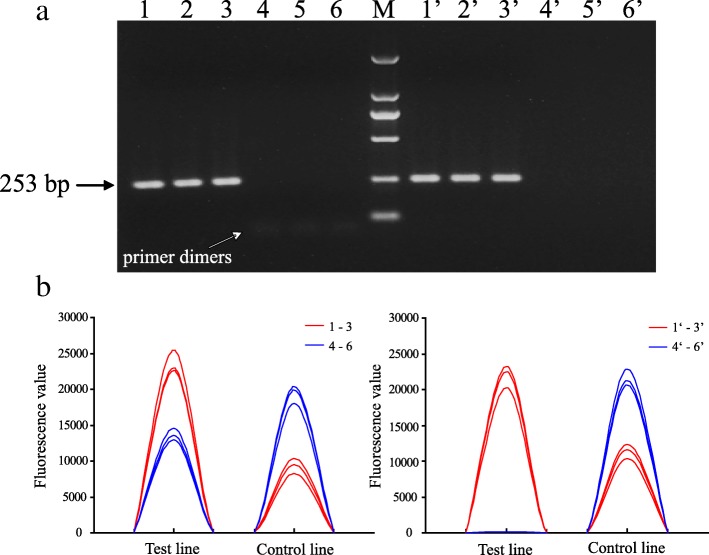
The establishment of PCR-LFIA for CPV-2 detection. **a** PCR result determined by 1.5% agarose gel electrophoresis. Lane M, DL-2000 DNA marker. Lane 1–3, positive control CPV-2-N1. Lane 4–6, negative control. Lane 1′-6′, PCR products of positive and negative control purified by Magbeads PCR Purification Kit, respectively. **b** Corresponding fluorescence signals of test and control lines read by Nanoeasy 1700 for the same samples as in Fig. 3a

To establish the optimal conditions for PCR-LFIA, positive control (CPV-2-N1 strain) and negative control (PBS) were used for determining both amplified products and primer dimers and demonstrating the effect of PCR products purification (Fig. 3; Table 1). As shown in Fig. 3, the PCR-GE method confirmed a correct amplification target of 253-bp fragment. Only some primer dimers were observed in the negative control. In addition, the effect of PCR products purification was simultaneously verified by 1.5% agarose gel electrophoresis. The bands of positive control remained bright but the primer-dimer bands disappeared in negative control (Fig. 3a). Furthermore, the results of PCR-LFIA were then confirmed by Nanoeasy 1700. Correspondingly, the positive control displayed a specific characteristic peak of test line and showed a slight decrease with purification. However, the negative control showed no specific characteristic peak and its fluorescence value was significantly reduced with purification (below the test strip’s cutoff value of 62) (Fig. 3b). The results verified the PCR method and the effect of purifying PCR products based on magnetic beads, which would effectively overcome the obstacle of false positive results.

**Table 1:** Fluorescence signal values of PCR products and purified PCR products measured by Nanoeasy 1700

Samples	PCR products		Samples	Purified PCR products	
	T	C		T	C
1	25,393	8237	1’	23,197	10,351
2	23,016	9562	2’	22,459	11,623
3	22,589	10,351	3’	20,264	12,362
4	13,623	19,862	4’	102	21,292
5	14,522	18,037	5’	118	20,634
6	12,985	20,351	6’	95	22,835

### Specificity of PCR-LFIA for detection of CPV-2

**Fig. 4:**
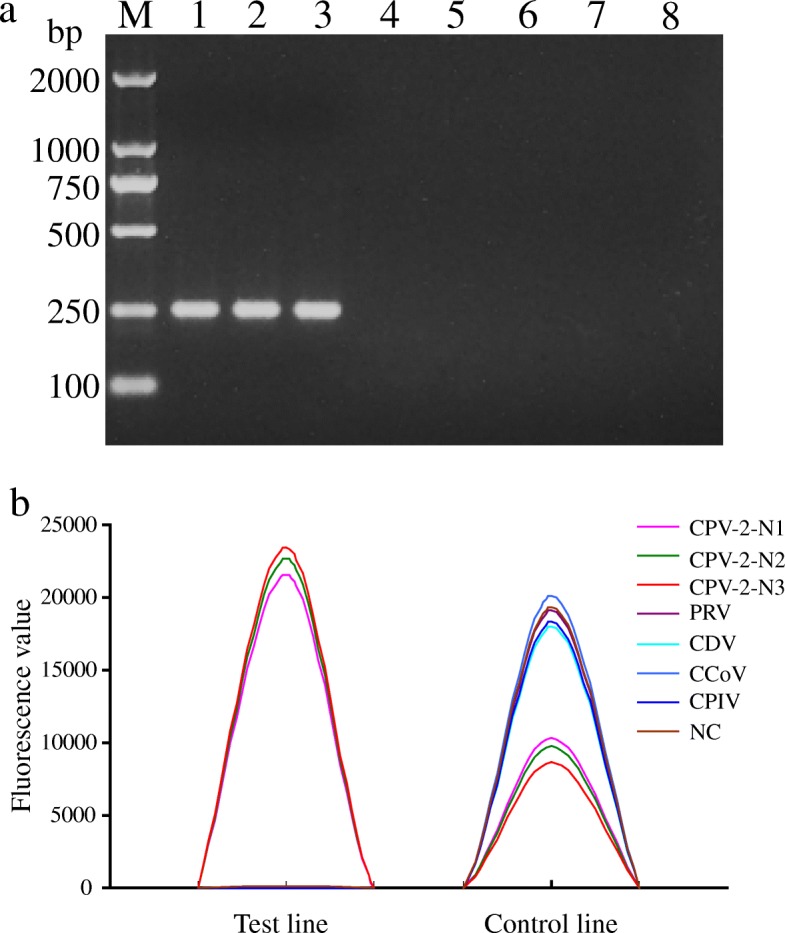
Specificity assessment of the PCR-LFIA for CPV-2 detection. **a** Specificity of PCR assay performed by 1.5% agarose gel electrophoresis. Lane M, DL-2000 DNA marker. Lane 1–8, CPV-2-N1, CPV-2-N2, CPV-2-N3, PRV-R1, CDV-NJ2, CCoV-C5, CPIV-J2, negative control, respectively. **b** Corresponding fluorescence signals of test and control lines for the same samples as in Fig. 4a

In order to evaluate the specificity of PCR-LFIA, potential cross-reactions were performed using DNA/RNA three positive strains (CPV-2-N1, CPV-2-N2, and CPV-2-N3) and several non-CPV strains (PRV-R1, CDV-NJ2, CCoV-C5, CPIV-J2) were tested in this study. PCR products were analyzed by 1.5% agarose gel electrophoresis and LFIA. As shown in Fig. 4a, cross-amplification tests using templates from PRV, CDV, CCoV and CPIV showed that no amplicons were detected, whereas the reaction using the CPV-2 template gave a positive result. The quantitative results were also obtained by Nanoeasy 1700 (Table 2), and the specific characteristic peak appeared only for CPV-2 detection (Fig. 4b). These results indicated that the PCR-LFIA developed in this study was specific for CPV-2.

**Table 2:** Fluorescence signal values measured by Nanoeasy 1700

	CPV-2-N1	CPV-2-N2	CPV-2-N3	PRV-R1	CDV-NJ2	CCoV-C5	CPIV-J2	NC*
T	21,543	22,685	23,391	21	65	109	31	103
C	10,359	9736	8702	19,063	18,055	20,165	18,341	19,352

*NC indicates negative control

### Sensitivity of PCR-LFIA and PCR-GE for detection of CPV-2

**Fig. 5:**
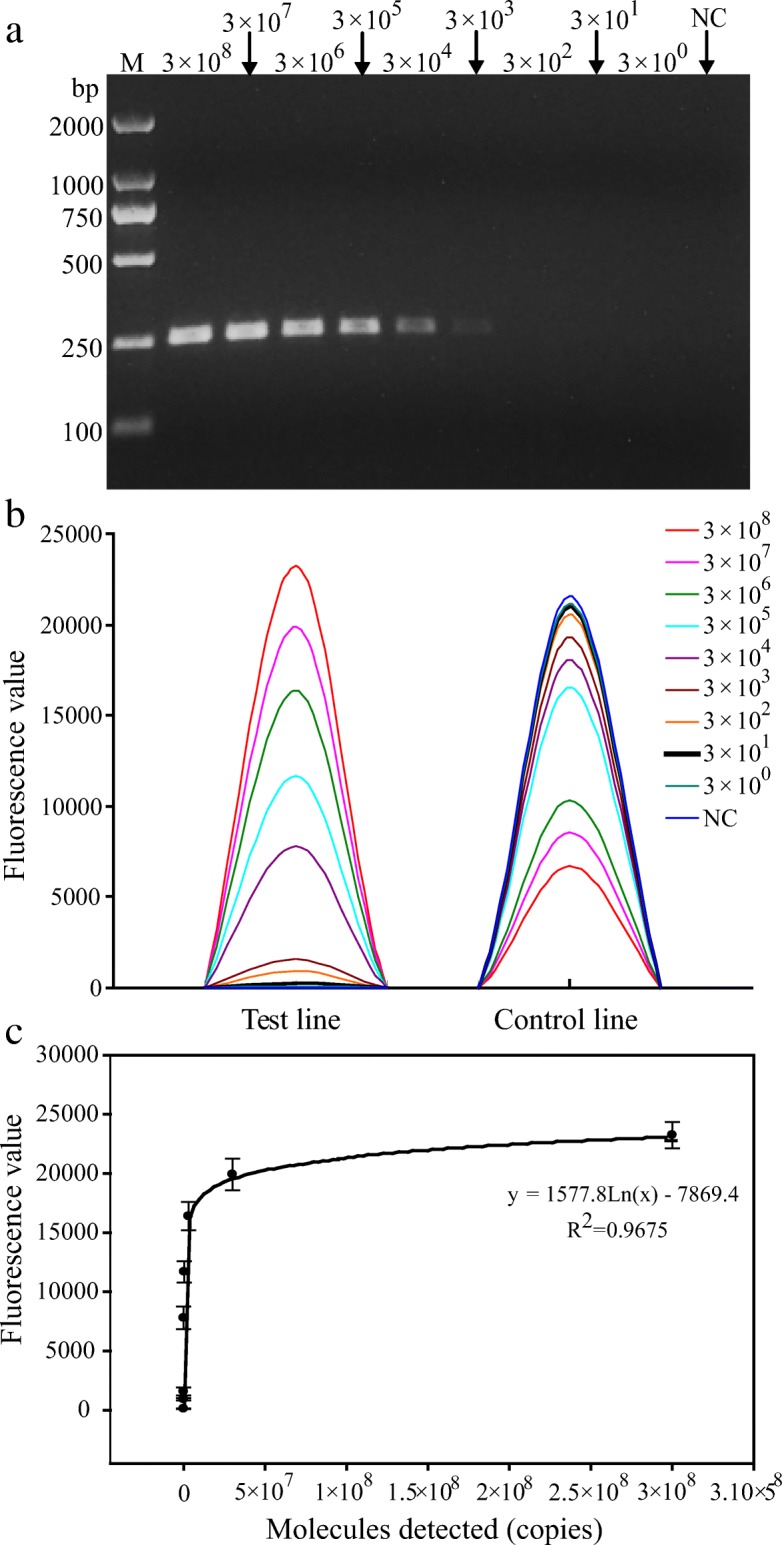
Sensitivity comparison of PCR-GE and PCR-LFIA for the detection of CPV-2. **a** Sensitivity of PCR-GE performed by 1.5% agarose gel electrophoresis. Lane M, DL-2000 DNA marker. Lane 2–10, 3 × 10^8^ copies/μL to 3 × 10^0^ copies/μL for standard DNA detected, respectively. Lane 11, negative control. **b** Corresponding signals of Test and Control lines for the same samples as in Fig. 5a. **c** Fluorescence signal values as a function of the copies of standard DNA detected. Each value was derived from three independent detections, and the error bars mean standard deviations

To determine the sensitivity of CPV-2 detection by PCR-GE and PCR-LFIA, the standard DNA stock (3 × 10^8^ copies/μL) was serially diluted tenfold. Each viral DNA (1 μL) was used as a template for PCR where the respective dilutions were subjected to thermal cycling using the primer pair of biotin-labeled CPV-2-F and Digoxigenin-labeled CPV-2-R, which amplified a 253-bp fragment from the VP2 gene of CPV-2. In PCR-LFIA, standard DNA dilutions gave a fluorescence signal values ranging from 131 to 23,251, and a linear relationship (y = 1577.8Ln(x)-7869.4, R^2^ = 0.9675) was observed with CPV-2 titers decreasing from 3 × 10^8^ to 3 × 10^0^ copies/μL. The results showed that the sensitivity of PCR-LFIA for the detection of CPV-2 was 3 × 10^1^ copies/μL, which was 100 times more sensitive than PCR-GE assay (Fig. 5; Table 3).

**Table 3:** Fluorescence signal values as a function of the copies of standard DNA measured by Nanoeasy 1700

Copies	3 × 10^8^	3 × 10^7^	3 × 10^6^	3 × 10^5^	3 × 10^4^	3 × 10^3^	3 × 10^2^	3 × 10^1^	3 × 10^0^	NC*
T_1_	23,256	21,439	17,285	12,643	6802	1878	1031	165	28	0
T_2_	22,138	18,948	16,859	10,853	8701	1206	929	93	66	11
T_3_	24,359	19,386	15,032	11,603	7902	1650	815	135	45	23
^−^X	23,251	19,924	16,392	11,700	7802	1578	925	131	46	11
SD	1111	1330	1197	899	953	342	108	37	19	12

*NC indicates negative control

### PCR-LFIA for detection of CPV-2 in clinical samples

**Fig. 6:**
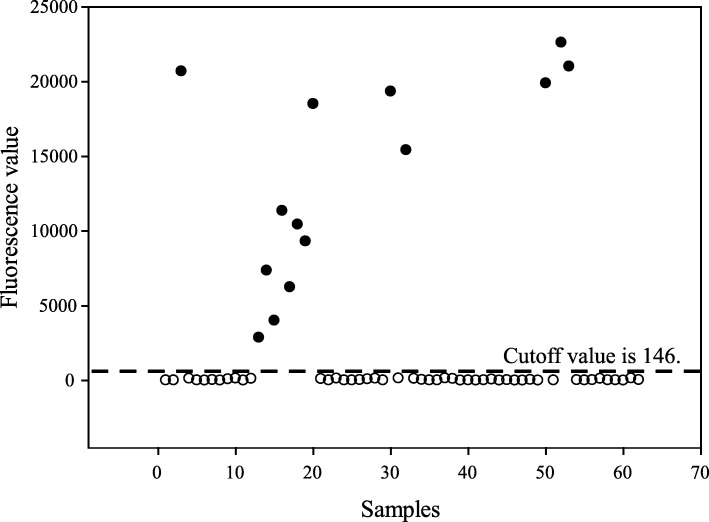
Fluorescent signals of clinical samples detected by the PCR-LFIA. “●” and “○” indicates positive and negative results, respectively

**Fig. 7:**
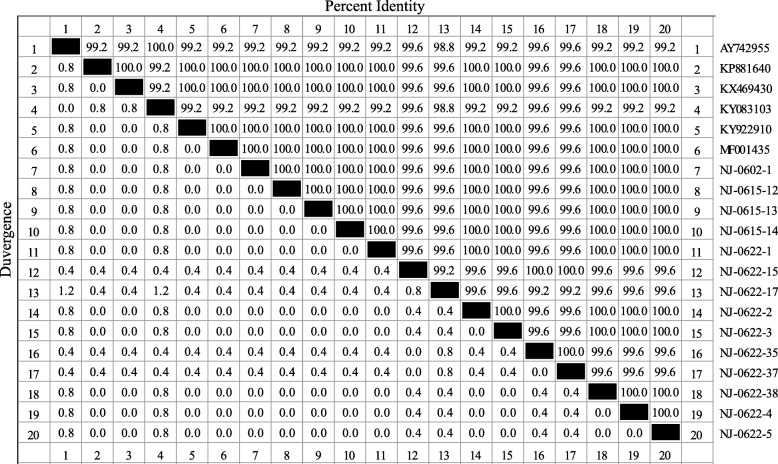
Identity and divergence analysis of partial VP2 gene in CPV-2 isolates. The partial VP2 nucleotide sequences from fourteen CPV-2 isolates were sequenced and analyzed by using ClustalW method with the Lasergene MegAlign software program for percent identity and divergence. The percent sequence identity was between 99.2% ~ 100%

Of the clinical samples, 22.6% (14 of 62) were determined to be CPV-2 positive by the PCR-LFIA. The cutoff value (the mean value plus 3× the standard deviation for negative samples) of LFIA is 146 (Fig. 6). The further analysis demonstrated the PCR-LFIA had a diagnostic agreement of 100% with PCR-GE (Table 4). All positive results were further confirmed by DNA sequencing to make sure that these are true positive isolates and there are no false-positive results. Sequences were edited using the SeqMan program and further verified by BLAST alignment tool (http://blast.ncbi.nlm.nih.gov/Blast.cgi). These sequences of CPV-2 isolated from other countries or regions such as USA, New Zealand, India, Singapore, Shanghai and Yangzhou (available through GenBank) were used for comparison with the fourteen CPV-2 isolates. Multiple-sequence alignments were constructed by using ClustalW method with the Lasergene MegAlign (DNASTAR, Madison, USA) software program (Fig. 7). All sequences of fourteen positive samples were submitted to National Center for Biotechnology Information databases (GenBank accession numbers: MH614361-MH614374).

**Table 4:** Clinical sample information and test result comparison for PCR-GE, PCR-LFIA and DNA sequencing

S. no.	Sample	Age (months)	Sex	Breed	Clinical suspicion	PCR-GE	PCR- LFIA	Signals	Antigenic type	Accession number
1	SG-01	2	F	French Bulldog	–	–	–	11		
2	SG-02	3	M	Pembroke Welsh Corgi	–	–	–	5		
3	NJ-0602-1	2.5	F	Border Collie	+	+	+	20,685	2c	MH614361
4	NJ-0602-2	3	F	Golden Retriever	–	–	–	122		
5	NJ-0602-3	12	M	Pembroke Welsh Corgi	–	–	–	9		
6	NJ-0602-4	12.5	F	Shiba Inu	–	–	–	0		
7	NJ-0615-01	4	M	Puppies	–	–	–	32		
8	NJ-0615-02	12	F	Pembroke Welsh Corgi	–	–	–	0		
9	NJ-0615-05	8	M	Pembroke Welsh Corgi	–	–	–	80		
10	NJ-0615-06	2	F	Samoyed	–	–	–	136		
11	NJ-0615-10	2	F	Bichon Frise	–	–	–	1		
12	NJ-0615-11	2	M	Teddy Bear	–	–	–	121		
13	NJ-0615-12	2.5	F	Golden Retriever	+	+	+	2865	2a	MH614362
14	NJ-0615-13	2	F	Pug	+	+	+	7354	2a	MH614363
15	NJ-0615-14	2	F	Pug	+	+	+	4003	2c	MH614364
16	NJ-0622-1	1.5	M	Pembroke Welsh Corgi	+	+	+	11,345	2c	MH614365
17	NJ-0622-2	3	F	French Bulldog	+	+	+	6246	2c	MH614366
18	NJ-0622-3	2.5	M	Pembroke Welsh Corgi	+	+	+	10,431	2b	MH614367
19	NJ-0622-4	4	F	French Bulldog	+	+	+	9311	2c	MH614368
20	NJ-0622-5	4	F	Teddy Bear	+	+	+	18,505	2c	MH614369
21	NJ-0622-6	6	M	Newfoundland and Labrador	–	–	–	97		
22	NJ-0622-7	8	M	Bichon Frise	–	–	–	10		
23	NJ-0622-8	5	F	Bichon Frise	–	–	–	132		
24	NJ-0622-9	6	F	Shiba Inu	–	–	–	5		
25	NJ-0622-10	6	F	Alaskan Malamute	–	–	–	10		
26	NJ-0622-11	2	M	Pomeranian	–	–	–	31		
27	NJ-0622-12	2	M	Puppies	–	–	–	82		
28	NJ-0622-13	2.5	F	Pug	–	–	–	128		
29	NJ-0622-14	2	F	Teddy Bear	–	–	–	12		
30	NJ-0622-15	1.5	M	Samoyed	–	+	+	19,337	2a	MH614370
31	NJ-0622-16	4	F	Bichon Frise	–	+	+	138		
32	NJ-0622-17	3	M	Golden Retriever	–	+	+	15,414	2c	MH614371
33	NJ-0622-18	2	F	Pembroke Welsh Corgi	–	–	–	116		
34	NJ-0622-19	8	M	Miniature Schnauzer	–	–	–	41		
35	NJ-0622-20	1.5	F	Pembroke Welsh Corgi	–	–	–	7		
36	NJ-0622-21	3	F	Border Collie	–	–	–	10		
37	NJ-0622-22	3	M	Golden Retriever	–	–	–	143		
38	NJ-0622-23	3	M	Golden Retriever	–	–	–	105		
39	NJ-0622-24	4	F	Pembroke Welsh Corgi	–	–	–	0		
40	NJ-0622-25	2.5	M	Pembroke Welsh Corgi	–	–	–	6		
41	NJ-0622-26	2	F	French Bulldog	–	–	–	3		
42	NJ-0622-27	2	M	Pembroke Welsh Corgi	–	–	–	14		
43	NJ-0622-28	3.5	F	French Bulldog	–	–	–	70		
44	NJ-0622-29	4	F	Border Collie	–	–	–	0		
45	NJ-0622-30	5	M	Samoyed	–	–	–	23		
46	NJ-0622-31	3	M	Samoyed	–	–	–	0		
47	NJ-0622-32	2	F	Border Collie	–	–	–	3		
48	NJ-0622-33	2	F	Pug	–	–	–	41		
49	NJ-0622-34	2.5	F	Pug	–	–	–	0		
50	NJ-0622-35	1.5	M	Pembroke Welsh Corgi	–	+	+	19,881	2c	MH614372
51	NJ-0622-36	2	F	Pug	–	–	–	8		
52	NJ-0622-37	2.5	M	Pug	–	+	+	22,607	2a	MH614373
53	NJ-0622-38	3	M	Pug	–	+	+	21,010	2c	MH614374
54	XW-01	5	F	Samoyed	–	–	–	23		
55	XW-02	4	M	Samoyed	–	–	–	9		
56	JC-01	2.5	F	Teddy Bear	–	–	–	25		
57	JC-02	8	M	Miniature Schnauzer	–	–	–	113		
58	JC-03	10	M	Pembroke Welsh Corgi	–	–	–	21		
59	SG-0616	3.5	F	Golden Retriever	–	–	–	19		
60	SG-0618	2.5	F	Pug	–	–	–	0		
61	SG-0619	3	M	Samoyed	–	–	–	141		
62	SG-0619	3	M	Samoyed	–	–	–	35

## Discussion

Parvovirus infections in dogs have become a global problem. The clinical signs resemble other enteric diseases and hence rapid and early diagnosis of the condition has become ever more urgent. Traditional methods such as virus isolation and electron microscopy are time-consuming, less sensitive, and expensive [35, 36]. Serological tests could detect the antibody, but fail to detect the acute infection. The commercial SNAP test is commonly used for rapid detection of CPV-2 on site, but it was less sensitive than PCR-based assays because it does not amplify the detection target during the test [7–9, 37]. Haemagglutination and immunochromatographic tests are widely used and simple, but they are less sensitive and always require fresh samples [8, 36]. Hence, these tests are now replaced by molecular methods like PCR which has high specificity and sensitivity than the traditional methods [38, 39]. However, the necessity of expensive equipment and reagents (real-time PCR) or cumbersome electrophoresis equipment and condition (PCR-GE) restricts its wide application [40].

Being visual inspection not applicable, to provide a faster way to quantify the PCR results rather than gel electrophoresis, the first polymerase chain reaction combined with fluorescent lateral flow immunoassay (PCR-LFIA) based on magnetic beads for rapid detection of CPV-2 has been successfully developed in this study. The lateral flow immunoassay used nanoparticles as signal amplifier, which ensures the sensitivity and accuracy of this assay. The optimized PCR-LFIA only needs about 80 min from PCR step, which is faster than PCR-GE and free of expensive equipment and toxic reagents. Due to the PCR-LFIA does not need gel electrophoresis, it is time-saving and carcinogenic ethidium bromide does not have to be used. The PCR products can be easily detected by LFIA based on magnetic beads purification, which can reduce the total time for detection of CPV-2. Furthermore, the accuracy and specificity of the PCR-LFIA will be greatly improved because magnetic beads for PCR products purification are employed. It can effectively remove the pollution of oligonucleotides, primer dimers, salts and proteins in PCR products or enzymatic reaction solutions.

The PCR-LFIA and PCR-GE exhibit high detection efficiency, and the specificity and relevance of them were confirmed. The results obtained from this study revealed that the PCR-LFIA possesses a high specificity to CPV-2 by giving positive results for all tested isolates of CPV-2 while yielding a negative result for non-CPV strains. In the same reaction conditions, PCR-LFIA showed 100 times more sensitive than PCR-GE when 10-fold serial dilutions of standard DNA were used. The sensitivity of PCR-LFIA with standard DNA in this study was 3 × 10^1^ copies/μL. The sensitivity was also higher than conventional PCR and recombinase polymerase amplification assay [41, 42]. The verification result of PCR-LFIA showed a similar sensitivity to the previously reported qPCR and real-time recombinase polymerase amplification assay [15, 43], while the inexpensive equipment required for PCR-LFIA makes the latest valuable molecular test method, especially for resource limited setting. The PCR-LFIA saves the time required for detection, and is safe and reliable. With the LFIA reader Nanoeasy 1700, the results can be quantified and shared in real time via the internet, making it convenient for customers to communicate with doctors.

## Conclusion

In conclusion, the first quantitative fluorescent PCR-LFIA assay for CPV-2 detection was successfully established. It had the high sensitivity of 3 × 10^1^ copies/μL. Cutoff value is 146. The developed PCR-LFIA is a sensitive, rapid, simple and valuable tool for quantitative detection of CPV-2 for both research and diagnostic purposes. It can also serve as a suitable molecular detection tool to facilitate timely and effective pathogenic microorganism investigations and response.

## Abbreviations

AuNP: Gold nanoparticle
C: Control line
CCoV: Canine coronavirus
CDV: Canine distemper virus
CPIV: Canine parainfluenza virus
CPV: Canine parvovirus
CPV-2: Canine parvovirus 2
ELISA: Enzyme-linked immunosorbent assay
FPV: Feline panleukopenia virus
HA: Haemagglutination
IC: Immunochromatographic
LFIA: Lateral flow immunoassay
PBS: Phosphate buffer saline
PCR: Polymerase chain reaction
PCR-GE: Polymerase chain reaction based on agarose gel electrophoresis
PRV: Pseudorabies virus
qPCR: Quantitative polymerase chain reaction
T: Test line
VI: Virus isolation
VP2: Capsid protein
